# Triple dissociation of duration perception regulating mechanisms: Top-down attention is inherent

**DOI:** 10.1371/journal.pone.0182639

**Published:** 2017-08-08

**Authors:** Yong-Jun Lin, Shinsuke Shimojo

**Affiliations:** Computation and Neural Systems, Division of Biology, California Institute of Technology, Pasadena, California, United States of America; Centre de neuroscience cognitive, FRANCE

## Abstract

The brain constantly adjusts perceived duration based on the recent event history. One such lab phenomenon is subjective time expansion induced in an oddball paradigm (“oddball chronostasis”), where the duration of a distinct item (oddball) appears subjectively longer when embedded in a series of other repeated items (standards). Three hypotheses have been separately proposed but it remains unresolved which or all of them are true: 1) attention prolongs oddball duration, 2) repetition suppression reduces standards duration, and 3) accumulative temporal preparation (anticipation) expedites the perceived item onset so as to lengthen its duration. We thus conducted critical systematic experiments to dissociate the relative contribution of all hypotheses, by orthogonally manipulating sequences types (repeated, ordered, or random) and target serial positions. Participants’ task was to judge whether a target lasts shorter or longer than its reference. The main finding was that a random item sequence still elicited significant chronostasis even though each item was odd. That is, simply being a target draws top-down attention and induces chronostasis. In Experiments 1 (digits) and 2 (orientations), top-down attention explained about half of the effect while saliency/adaptation explained the other half. Additionally, for non-repeated (ordered and random) sequence types, a target with later serial position still elicited stronger chronostasis, favoring a temporal preparation over a repetition suppression account. By contrast, in Experiment 3 (colors), top-down attention was likely the sole factor. Consequently, top-down attention is necessary and sometimes sufficient to explain oddball chronostasis; saliency/adaptation and temporal preparation are contingent factors. These critical boundary conditions revealed in our study serve as quantitative constraints for neural models of duration perception.

## Introduction

Perceiving time is special to humans because time itself is not a physical entity commensurate with matter and energy. Accordingly, the subjective sense of time may rely upon inferences from primary senses as well as a person’s cognitive state. In the sub-second range of time perception, a growing body of research has shown that the perceived duration of an event not only depends on its temporal context but also on the observer’s psychological activities. For example, in a string of events, humans tend to observe particular events as lasting longer, such as the first or the last in the sequence [1], or when there is an odd event in the context (e.g., [2]). The subjective expansion of time is also experienced when we switch hearing from one ear to the other [3], during the first moment after we shift our gaze to read a clock (e.g., [4]), and during the moment right before baseball or tennis players strike the ball [5]. These reported subtypes of subjective time expansion effects may share related neural underpinnings, reflecting how the brain constructs and regulates perceived time.

Prior research has revealed that time perception involves multiple cortical and subcortical brain areas (e.g., [6–8]). A clear picture of how these areas interact is still lacking. The aforementioned phenomena thus provide a window into how the brain takes bottom-up, stimulus-driven and top-down, goal-driven factors both into account. In this study, we focus on the mechanisms of subjective time expansion with an oddball paradigm. It is a perceptual case that does not concern action-related factors, and arguably one of the most theorized and experimented case among related phenomena. In such a paradigm, the duration of a novel item (oddball) appears subjectively longer than that of other repeated items (standards) in a sequence [2]. For the sake of simplicity, we will refer to this specific phenomenon as ‘oddball chronostasis’ in the remainder of this article, following the generic term ‘chronostasis’ first introduced by Yarrow et al. [4] to describe the situation that the second hand of a clock seems to have stopped (i.e., subjective time expansion).

In the literature, two major theories were proposed to explain oddball chronostasis that resonates with this top-down and bottom-up distinction. One is the attention theory, which states that attention increases the subjective duration of the oddball [2, 9]. The other is the repetition suppression theory, which instead states that through adaptation or predictability, the subjective duration of the standards reduces [10–13]. By contrast, the oddball stimulus appears longer because it is less susceptible to the repetition suppression.

Several previous key findings support the attention hypothesis. First, Tse et al. [2] discovered that the amount of visual duration distortion in general rose with reference duration with an initial local peak around 225 msec followed by a dip around 375 msec until it saturated beyond 1000 msec, consistent with the signature of transient and sustained components of attention (e.g., [14–15]). Given that attention orienting takes time, the attention hypothesis could also explain why anti-chronostasis (subjective time compression) occurred when the reference duration was less than about 120 ms. Second, Tse and colleagues [2] found that oddball chronostasis in visual and auditory cases shares similar patterns, which is consistent with a central mechanism account such as attention. Third, New & Scholl [9] found that the occurrence of an oddball dilated the perceived duration of another item simultaneously presented at a different location and the effect was unaffected by target-oddball spatial distance or whether the oddball and the target item belong to the same object. They thus concluded that this global time expansion effect might reflect increased arousal driven by attention.

On the other hand, other existing evidences support the repetition suppression hypothesis more. First, oddball chronostasis scales with oddball’s serial position [12, 16–17]. Second, oddball chronostasis scales with feature discrepancy between the oddball and the standards [12–13, 16]. Third, oddball chronostasis can be elicited by brightness oddballs dimmer than the standards [13]. Therefore, the underlying mechanisms of repetition suppression may involve low-level adaptation as well as high-level prediction error of stimulus content or position.

Interestingly, the attention and the repetition suppression hypotheses are in fact logically compatible. The former predicts the subjective expansion of the oddball duration while the latter subjective compression of the standard duration. It means that these two hypotheses can both possibly contribute to the oddball chronostasis, and that they can be directly compared in a single experiment. First, the attention effect can be broken down into bottom-up saliency and top-down attention (c.f. [2]). Second, the repetition suppression effect can be divided into adaptation as well as prediction error [13]. Therefore, the four new hypotheses in question become top-down attention, bottom-up saliency, adaptation, and prediction error. Theoretically saliency and adaptation are different, but indistinguishable empirically in the operational definition of the following experiments. Accordingly, we regroup the hypotheses above into three instead: top-down attention, saliency/adaptation, and prediction error and aim to identify their relative contributions across experimental conditions.

**Fig 1:**
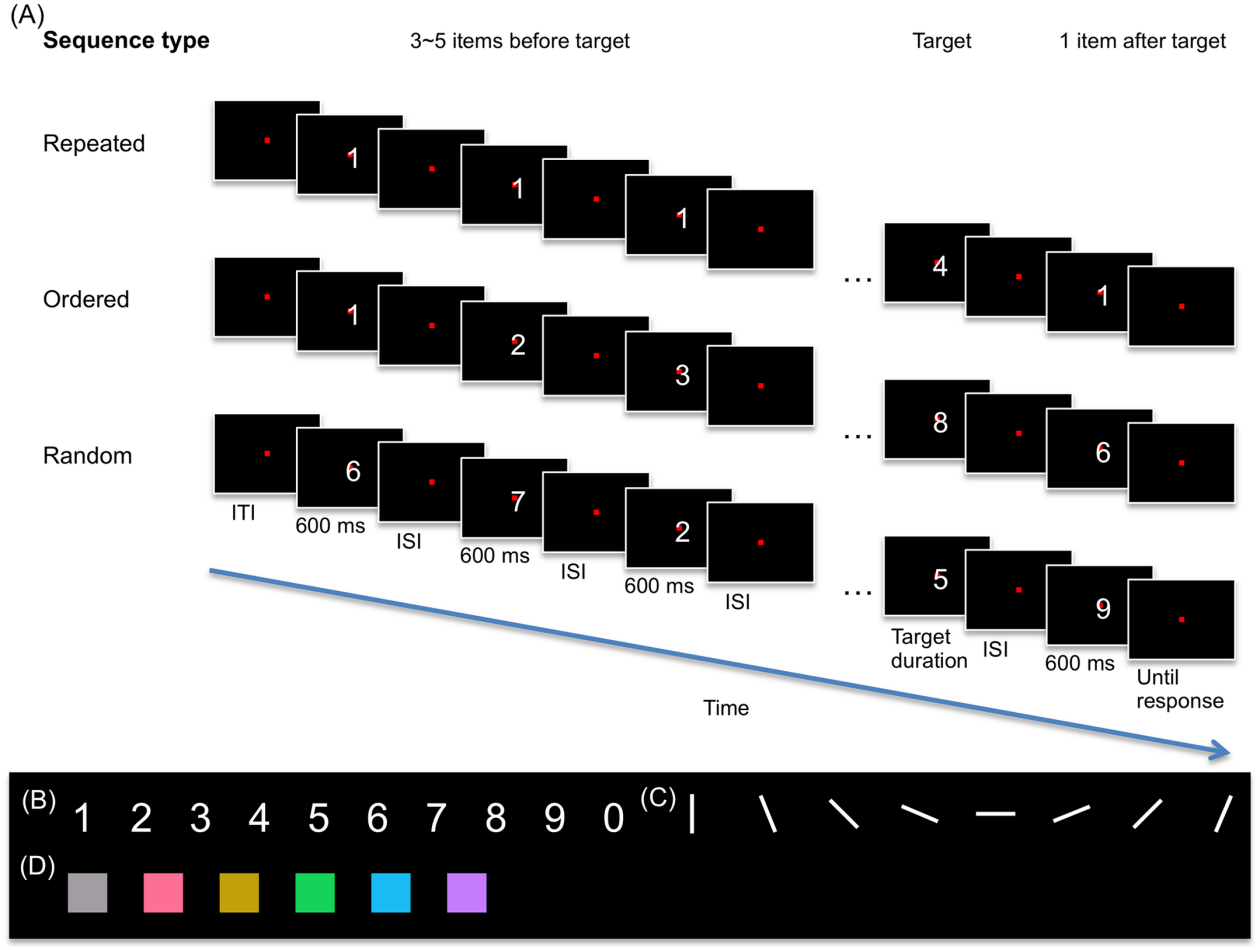
Paradigm and stimuli. (A) In each type of item sequences, the observers performed a duration discrimination task between the target and its reference to estimate the amount of duration distortion (see Methods for details). (B-D) Stimulus sets in Experiments 1, 2, and 3, respectively.

**Fig 2:**
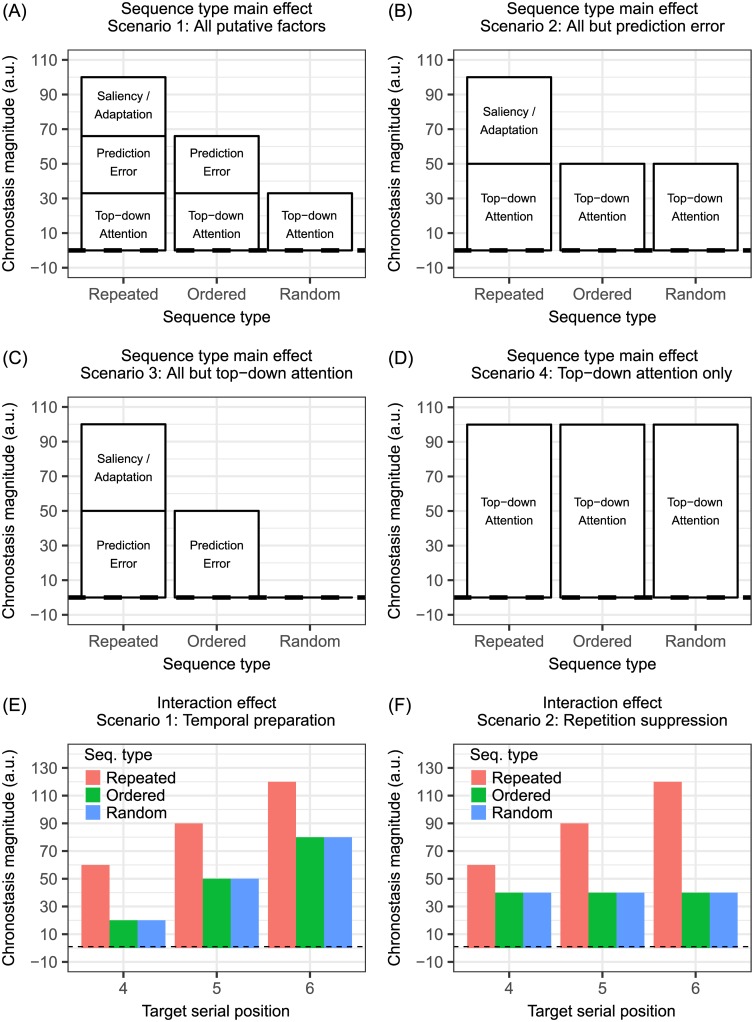
Theoretical predictions of results. Vertical axis is chronostasis magnitude (the amount of subjective time expansion, see Methods and supplementary methods for details). (A-D) Four likely scenarios of the sequence type main effect, given the controversy between the attention and the repetition suppression theories. (E-F) Two likely scenarios of the interaction effect between sequence types and target serial positions, given the controversy between temporal preparation and repetition suppression theories. See introduction for details of the rationale and the experimental design. The predictions here assume additive and independent components.

To dissociate these refined hypotheses, we measured participants’ chronostasis magnitude (CM, which quantifies how large the subjective time expansion of a target is when compared with a reference, see Materials and methods for details) with three types of item sequences: repeated, ordered, and random (Fig 1A). The repeated condition was the classic oddball paradigm. The standards were repeated and the oddball was distinct from the standards. As Fig 2A illustrates, in the most comprehensive scenario, top-down attention, saliency/adaptation, and prediction error could all be underlying factors. In the ordered condition, items never repeated; hence, this condition excluded adaptation/saliency as an explanation. The target was the item that did not follow the regularity. In the random condition, the observers were pre-instructed which item would be the target while the other items were random and carried no information about what the target would be. Compared with an ordered sequence, a random sequence further excluded prediction error and thus singled top-down attention out as an underlying cause. Therefore, contrasting CM across sequence types could unravel which factors are critical. Fig 2A–2D depict several possible scenarios of the sequence type effect.

Apart from the main issue regarding the origin of oddball chronostasis, there is also a side issue about what mechanism modulates it. Several previous studies [12, 16–17] have reported that the larger the serial position of the oddball is inside a sequence, the greater the CM, and the effect could eventually saturate. Two explanations have been proposed. One is the temporal preparation account by Kim and McAuley [16] which states that oddball chronostasis at least partly originates from how early one could detect the onset of the target item. Assuming that the offset is unaffected, an apparently earlier onset is tantamount to longer perceived duration. Using an oddball detection task, their data did reveal such a negative correlation between response time and CM. The other is the repetition suppression account [10–13] as described earlier, which states that the more the repetition, the greater the CM. However, in previous experiments [12, 16–17] reporting this target serial position effect, standards were always repeated. Thus, a critical test between these two hypotheses was missing. By contrast, in our design, the sequence type and the target serial position were orthogonally manipulated. If the temporal preparation hypothesis were true, CM would grow with target serial position regardless of sequence type (Fig 2E), resulting to no interaction effect. Conversely, if the repetition suppression hypothesis were true, CM would grow with target serial position in the repeated but not in the ordered and the random sequence condition, leading to significant interaction effect (Fig 2F).

In Experiment 1, we started from digits as stimuli (Fig 1B), as these distinct sequence types can be intuitively constructed (Fig 1A). The results demonstrated that half of the oddball chronostasis could be explained by top-down attention, while the other half by bottom-up saliency or adaptation, but not prediction error. To determine whether the findings can be generalized to lower level visual attributes, Experiment 2 tested orientations as stimuli, which were also adopted in several previous studies [10, 12–13]. To further disentangle adaptation from saliency, Experiment 3 examined colors because they are highly salient and draw stimulus-driven attention rather automatically. The results revealed that top-down attention is sufficient as an explanation for oddball chronostasis among colors. The overall implications for top-down and bottom-up mechanisms underlying chronostasis are discussed.

## Materials and methods

### Participants

All experiment protocols were approved by the institutional review board of California Institute of Technology (Caltech). The observers were drawn from the Caltech brain science subject pool, which mainly consisted of Caltech and Pasadena City of College students. In Experiment 1, there were 16 observers (8 female; 14 naïve; 20–54 years; M = 29.8; SD = 8.1). In Experiment 2, there were 19 participants (9 female; 18 naïve; 18–59 years; M = 27.5; SD = 10.5). In Experiment 3, there were 14 participants (6 female; 14 naïve; 18–41 years; M = 25.4; SD = 7.6). All participants provided written informed consent before the experiments. The naïve participants were paid for their cooperation. Participants of Experiment 3 all passed an Ishihara color blindness test.

### Apparatus

The stimuli were presented using a Philips Brilliance 202P7 monitor in Experiments 1 and 3, and a Lacie Electron 22 Blue IV monitor in Experiment 2. The screen resolution was set at 800 (H) x 600 (V) with frame rate 120Hz. The viewing distance was 70cm. The stimuli presentation code was written in Python with PsychoPy toolbox [18] under Mac OS X. To ensure that the timing was precise, a preemptive setting was adopted to override other background processes. All Commission Internationale d’Éclairage (CIE) 1931 chromaticity (x, y) and luminance (Y) values were measured by a Konica Minolta CS-100A colorimeter.

### Design

The two independent variables sequence type and target serial position were factorially crossed within subjects. There were 3 levels of sequence types (repeated, ordered, random) in Experiments 1 and 2, and 2 levels (repeated, random) in Experiment 3. There were 3 levels of target positions (4th, 5th, 6th) in all experiments. The dependent variable was chronostasis magnitude (CM), whose measurement is described in the later subsections. Therefore, CM was analyzed with a two-way analysis of variance (ANOVA) in each experiment.

### Stimuli

All stimuli were presented at the center of the screen where there was a constant red (CIE x = 0.614, y = 0.342, Y = 26.1) fixation point spanning 0.1° (H) x 0.1° (V) visual angle at the center of the screen. The background was black. In Experiment 1, the stimuli set comprised white digits 0 to 9 each spanning 1.0° (H) x 1.5° (V) visual angle (Fig 1B). In Experiment 2, the stimuli set comprised bars spanning 5° (H) x 1° (V) visual angle with one of the 8 orientations: 0°, 22.5°, 45°, 67.5°, 90°, 112.5°, 135° and 157.5° (Fig 1C). In the ordered condition, the orientation sequence followed either a clockwise or a counter-clockwise order, where the step between stimuli was either -22.5° or 22.5°. The target was the item that deviated from the expected regularity by skipping ahead or going backwards. To remain perceptual consistency, the last item resumed the regularity from the target rather than the items preceding the target. In Experiment 3, the stimuli set comprised squares spanning 4° (H) x 4° (V) visual angle with one of the 6 nominally isoluminant colors: red (x = 0.39, y = 0.31, Y = 61.7), yellow (x = 0.43, y = 0.47, Y = 61.7), green (x = 0.26, y = 0.47, Y = 61.7), blue (x = 0.20, y = 0.28, Y = 61.7), purple (x = 0.29, y = 0.24, Y = 61.7), and gray (x = 0.31, y = 0.33, Y = 61.7) (Fig 1D). Unlike Experiments 1 and 2, Experiment 3 did not include an ordered condition since there was not a clear way to order the colors. The item colors were specifically chosen to be isoluminant because it was known that stimulus luminance could affect its perceived duration (e.g., [19]).

### Procedure

The dependent variable CM was derived from the psychometric function estimated by the method of constant stimuli (see the subsection below for details), Thus, the participants’ task was to discriminate the duration between the target item and its reference, which was the item just before it (Fig 1A). They were forced to make their best guess and to choose either target perceived “longer” or “shorter” using one of two keys on the computer keyboard.

In each trial, 5 to 7 items were presented one at a time, as the target serial position was between the 4th and the 6th, and an additional item was presented after the target (Fig 1A). We specifically avoided using the first or the last item as the target or the reference to avoid the potential confounding chronostasis effects elicited by the first and the last stimulus (e.g., [1]), as in van Wassenhove and colleagues’ oddball chronostasis study [20]. The participants were also instructed to respond only after all items had been presented. This long gap between the target item presentation and the response helped avoid chronostasis induced by action preparation (e.g., [5]). Duration of all items except the target was fixed at 600 ms. Centered around that standard duration, the target duration varied in 8 levels from 250 to 950 ms in 100 ms steps to avoid inflation of CM caused by asymmetric distribution [21]. Each inter-stimulus interval (ISI) was random, chosen from a uniform distribution between 250 and 500 ms. Each inter-trial interval (ITI) was random, chosen from a uniform distribution between 700 and 1100 ms.

Every participant was tested in 3 sessions, where each session comprised a practice block and 10 blocks of trials of a given sequence type. The order of sequence types was random per participant. A session typically took about 40 minutes. The participants were always given two separate days to finish all 3 sessions. Each session had a sequence type specific experimental instruction, followed by a practice block of 6 trials, and experiment blocks (10 in Experiments 1 and 2; 9 in Experiment 3). The practice trials were from the conditions of shortest (250 ms) and longest (950 ms) target durations and were excluded from analysis. Each of the experiment blocks contained one repeat of all 24 combinations of 3 target serial position and 8 target durations, whose order was randomly shuffled. Before each block, an instruction screen indicated the chance to take a short break.

The actual items per trial depended on the sequence type (Fig 1A). The repeated condition was the classical oddball paradigm, where all items except the target were the same. Hence, the oddball was the target. All items were randomly redrawn from the stimulus set per trial. In the ordered condition, the sequence always was ascending and started from 1. The target was the item that deviated from the expected regularity. In the random condition, the target item was randomly chosen from the stimulus set per block and prompted in the block instruction. Each of the standard items was sampled with replacement from the stimulus set excluding the target stimulus.

### Psychometric function and chronostasis magnitude

In each sequence type and target serial order combination, the probability of a participant reporting the target as subjectively longer than the reference were calculated. A cumulative Gaussian distribution was fitted to those data to estimate psychometric functions. The point of subjective equality (PSE) of each condition was the corresponding physical duration when the probability was 0.5 on the psychometric function. Chronostasis magnitude was thus defined as CM = reference duration—PSE. For example, a 500 ms target subjectively equivalent to a 600 ms reference this way indicates CM of 100 ms.

## Results

### Experiment 1: Digits

Crossing the factors sequence type and target serial position resulted in 9 conditions per participant. A two-way 3 (sequence type) x 3 (target position) repeated measures ANOVA was performed on the measured CM. Our refined hypotheses predict that the sequence type main effect on CM could fall into one of the four scenarios as in Fig 2A–2D, resolving which of the top-down attention, saliency/adaptation, and prediction error hypotheses are true. The interaction effect between these two factors could also fall into either scenario as in Fig 2E and 2F, suggesting whether the temporal preparation or the repetition suppression hypothesis is more likely.

**Fig 3:**
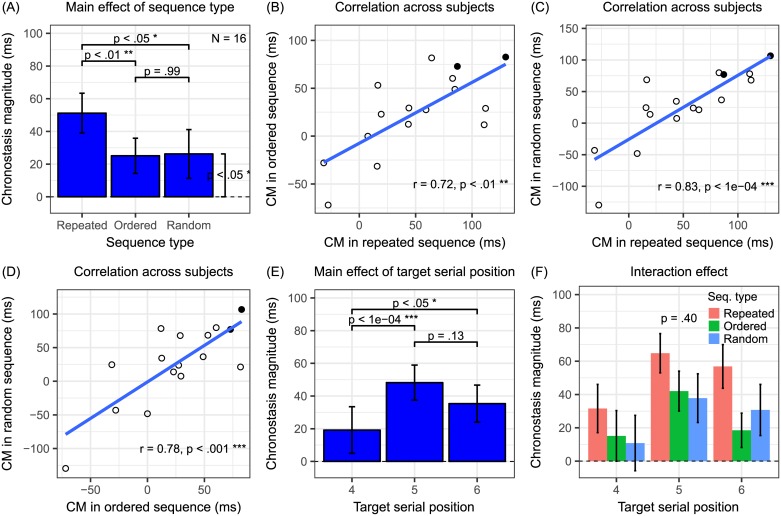
Results from Experiment 1 (digits). (A, E, F) The group average CM bar graphs along with statistical outcomes. Error bars indicate 1 S.E.M. (A) The significant sequence type main effect is consistent with the prediction scenario shown in Fig 2B. (B-D) Scatter plots and correlations of CM between sequence type condition pairs. Each data point represents one participant. (E) The significant target serial position main effect is consistent with previous findings. (F) The non-significant interaction effect of sequence type and target serial position matches closer to the data pattern predicted in Fig 2E.

Fig 3 summarizes the results (N = 16) from Experiment 1. The main effect of sequence types was significant (*F*_2,30_ = 5.42, *P* < 0.01, **η**_**p**_^**2**^ = 0.27) (Fig 3A). Tukey post-hoc tests revealed that CM significantly differed between the repeated and the ordered conditions (*Z*_1,30_ = 3.01, *P* < 0.01), between the repeated and the random conditions (*Z*_1,30_ = 2.78, *P* < 0.05), but not between the ordered and the random conditions (*Z*_1,30_ = -0.22, *P* = 0.97). Since in the random sequence condition, the block-specific targets were defined at the beginning of each block, to verify that participants correctly memorized the target while performing the task, every block was broken into two halves. PSEs derived from the first and the second halves were compared with a two-tailed paired t-test. The non-significant difference (t_15_ = 0.92, *P* = 0.37) suggested that participants were able to perform the task with robust memory of the targets. A one-tailed paired t-test further showed that CM in the random condition significantly differed from 0 (t_15_ = 1.76, *P* < 0.05). The data pattern matched the prediction in Fig 2B. Therefore, it suggests that both top-down attention and saliency/adaptation, but not prediction error, are the necessary underlying factors of oddball chronostasis at least for digits stimuli. The exclusion of prediction error was in line with the conclusion of Cai and colleagues [10] that high-level stimulus content prediction may not be an underlying factor. Our experimental design and data further showed that top-down attention and saliency/adaptation contributed about equally. Pearson’s correlation of CM across participants between sequence types also strengthened this conclusion. The correlation was significant between the repeated and the ordered conditions (r = 0.72, *P* < 0.01), between the repeated and the random conditions (r = 0.83, *P* < 10^−4^), and between the ordered and the random conditions (r = 0.78, *P* < 0.001) (Fig 3B–3D). It suggests that chronostasis among those three sequence types share a common mechanism, which is likely top-down attention.

The main effect of target serial position was significant (*F*_2,30_ = 9.45, *P* < 0.001, **η**_**p**_^**2**^ = 0.39) (Fig 3E). The Tukey post-hoc tests of target serial positions revealed that CM significantly differed between the 4th and the 5th (*Z*_1,30_ = -4.34, *P* < 0.001), between the 4th and the 6^th^ (*Z*_1,30_ = -2.42, *P* < 0.05), but not between the 5th and the 6th positions (*Z*_1,30_ = 1.92, *P* = 0.13). The pattern was consistent with the results of experiment 3 in Pariyadath and Eagleman [12] that CM grows with the oddball position but eventually saturates.

Unlike the prediction of the repetition suppression hypothesis (Fig 2F), the interaction effect was not significant (*F*_2.65,39.82_ = 1.00, *P* = 0.40, **η**_**p**_^**2**^ = 0.06, Greenhouse-Geisser correction applied) (Fig 3F). Although the data pattern seemed to match the prediction of the temporal preparation hypothesis (Fig 2E), a planned comparison could better reveal the source of this non-significant interaction. If the temporal preparation hypothesis were really true, a later target would elicit larger CM no matter the sequence type. That is, even in the non-repeated (ordered and random) sequences, CM would be larger with a later target. A planned one-tailed t-test, averaging CM in the ordered and the random conditions, showed that this was indeed the case with the most extreme contrast (6th vs. 4th target serial position: t_60_ = 1.88, *P* < 0.05). Therefore, the results here favored the temporal preparation [16] over the repetition suppression hypothesis [12, 17] in explaining target serial position effect.

To our knowledge, this is the first systematic experiment directly contrasts the attention with the repetition suppression hypotheses, as well as the temporal preparation with the repetition suppression hypotheses. The results imply that both top-down attention and saliency/adaptation are necessary for explaining oddball chronostasis among digits. On top of that, temporal preparation rather than repetition suppression separately modulates the chronostasis effect.

### Experiment 2: Orientations

One may argue that the results in Experiment 1 are specific to digits, which require more cognitive processing than basic perceptual attributes. In Experiment 2, we extended the same paradigm to stimuli varied in orientations to examine if the conclusion holds.

**Fig 4:**
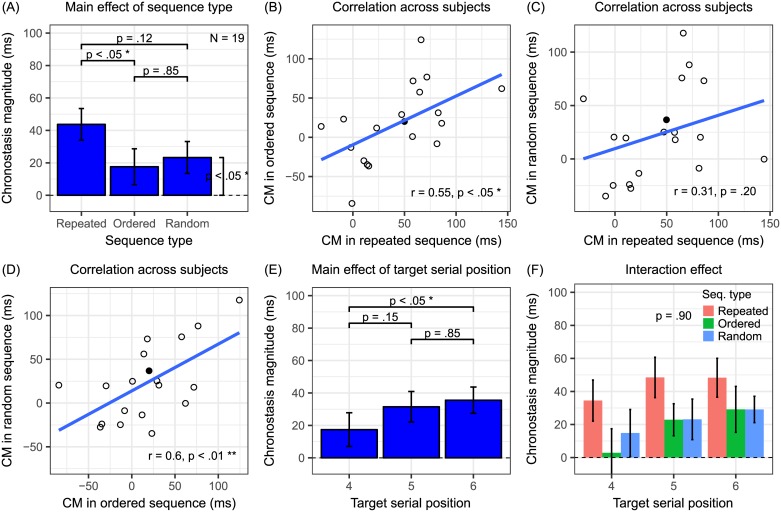
Results from Experiment 2 (orientations). The data patterns basically replicate those in Experiment 1 (Fig 3). See Fig 3 caption for other details.

We obtained a similar pattern in Experiment 2. Fig 4 summarizes the results (N = 19) from Experiment 2. The main effect of sequence types was significant (*F*_2,36_ = 3.45, *P* < 0.05, **η**_**p**_^**2**^ = 0.16) (Fig 4A). Tukey post-hoc tests revealed that CM significantly differed between the repeated and the ordered conditions (*Z*_1,36_ = 2.52, *P* < 0.05), but not between the repeated and the random conditions (*Z*_1,36_ = 1.97, *P* = 0.12), or between the ordered and the random conditions (*Z*_1,36_ = -0.55, *P* = 0.85). To verify that participants performed the task with stable memory of the block-specific target in the random sequence condition, PSEs derived from the first and the second halves across all blocks were compared with a two-tailed paired t-test. The non-significant difference (t_18_ = 0.82, *P* = 0.42) suggested that participants had robust memory of the target. A one-tailed t-test further showed that CM in the random condition significantly differed from 0 (t_18_ = 2.37, *P* < 0.05). The data pattern was again consistent with the prediction in Fig 2B. Pearson’s correlation of CM across participants between sequence types also showed similarity. The correlation was significant between the repeated and the ordered conditions (r = 0.55, *P* < 0.05), between the ordered and the random conditions (r = 0.60, *P* < 0.01), but not between the repeated and the random conditions (r = 0.31, *P* = 0.20) (Fig 4B–4D). Compared with the results of Experiment 1, in general, the correlation coefficients were lower and one of them was not significant. The reason could be that specific bar orientations were not as overlearned items as digits, making the task harder and thus data pattern noisier than those in Experiment 1.

The main effect of target serial position was marginally significant (*F*_2,36_ = 3.22, *P* = 0.05, **η**_**p**_^**2**^ = 0.15) (Fig 4E). For the sake of comparison, the Tukey post-hoc tests of target serial positions were still performed, but one must take precaution in the interpretation. It revealed that the CM significantly differed between the 4th and the 6th (*Z*_1,36_ = -2.42, *P* < 0.05), but not between the 4th and the 5^th^ (*Z*_1,36_ = -1.88, *P* = 0.15), or the 5th and the 6th positions (*Z*_1,36_ = -0.54, *P* = 0.85).

The interaction effect was not significant (*F*_4,72_ = 0.27, *P* = 0.90, **η**_**p**_^**2**^ = 0.01) (Fig 4F). To find out whether the lack of interaction was due to significant target serial position effect in the non-repeated sequences, a planned one-tailed t-test averaging CM in the ordered and the random conditions revealed that CM in the 6th was larger than that in the 4th target serial position (t_72_ = 2.61, *P* < 0.01). Therefore, consistent with Experiment 1, the results here also supported the temporal preparation [16] over the repetition suppression hypothesis [12, 17] in explaining target serial position effect.

Therefore, consistent with Experiment 1, the results again imply that both top-down attention and saliency/adaptation are necessary factors of oddball chronostasis and they contribute about equally. Temporal preparation is a separate modulatory effect.

### Experiment 3: Colors

Although Experiments 1 and 2 both arrived at the same conclusion, it is still likely that the conclusion may not be generalizable to other stimulus attributes. This motivated us to carry out an experiment with nominally isoluminant color stimuli (see Materials and methods for details), which was, to our knowledge, never examined in the oddball chronostasis literature. Due to the difficulty of ordering colors, only the repeated and the random sequences were included. The results revealed a distinct pattern of the sequence type effect, while everything else were qualitatively similar to those in Experiments 1 and 2.

**Fig 5:**
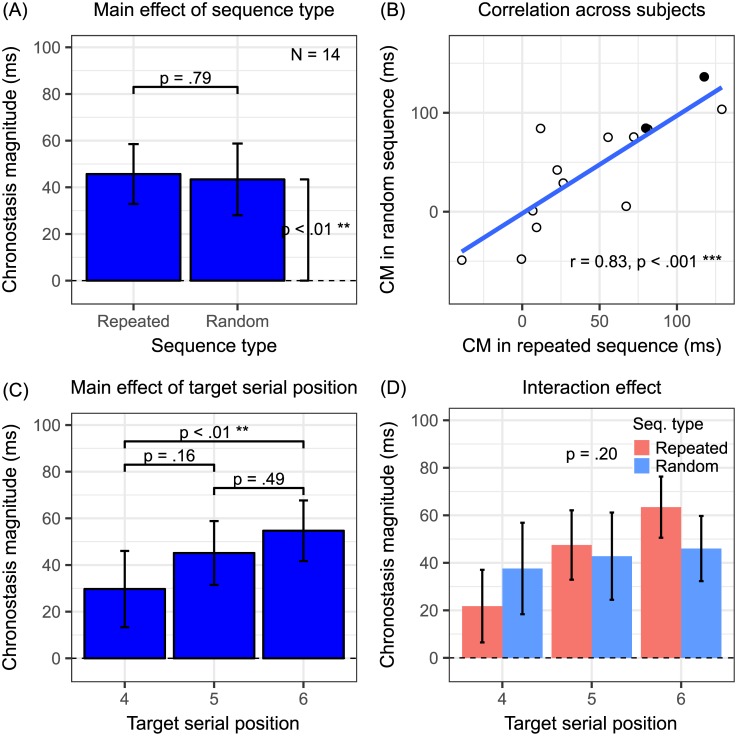
Results of Experiment 3 (colors). There are only repeated and random sequence type conditions. (A) The non-significant sequence type main effect is consistent with the prediction scenario shown in Fig 2D. (B-D) Despite the stark difference in sequence type main effect from Experiments 1 and 2, other patterns remain similar to those in the first two experiments. See Fig 3 caption for other details.

Fig 5 summarizes the results (N = 14) from Experiment 3. The main effect of sequence types was not significant (*F*_1,13_ = 0.07, *P* = 0.80, **η**_**p**_^**2**^ = 0.01) (Fig 5A). PSEs separately derived from the first and the second halves across all random sequence blocks were compared with a two-tailed paired t-test. The non-significant difference (t_13_ = 1.81, *P* = 0.09) suggested that the participants were able to perform the task with stable memory of the block-specific targets. A one-tailed t-test further showed that CM in the random condition significantly differed from 0 (t_13_ = 2.82, *P* < 0.01). The data pattern matched the prediction in Fig 2D instead. Therefore, it may indicate that top-down attention but not saliency/adaptation or prediction error, is a sufficient underlying factor of oddball chronostasis at least for isoluminant color stimuli. Pearson’s correlation of CM across participants between the repeated and the random conditions was significant (r = 0.83, *P* < 0.001) (Fig 5B). Taken together, it suggests that the common mechanism underlying the chronostasis of both sequence types is likely top-down attention.

The rest was mostly similar to Experiments 1 and 2. The main effect of target serial position was significant (*F*_2,26_ = 4.46, *P* < 0.05, **η**_**p**_^**2**^ = 0.26) (Fig 5C). The Tukey post-hoc tests of target serial positions revealed that CM significantly differed between the 4th and the 6th (*Z*_1,26_ = -2.96, *P* < 0.01), but not between the 4th and the 5^th^ (*Z*_1,26_ = -1.83, *P* = 0.16), or the 5th and the 6th positions (*Z*_1,26_ = -1.13, *P* = 0.49). The interaction effect was not significant (*F*_2,26_ = 1.72, 1144, *P* = 0.20, **η**_**p**_^**2**^ = 0.12) (Fig 5D). However, considering only the random sequence type, a planned one-tailed t-test did not reveal larger CM in the 6th than that in the 4th target serial position (t_26_ = 0.02, *P* = 0.25). Therefore, the results here could not directly distinguish between the temporal preparation and the repetition suppression hypothesis. As the exact isoluminant colors were harder to remember compared with the exact digits or orientations, one possible explanation was that temporal preparation might rely on unambiguous memory of the target stimulus.

It is intriguing as to why the sequence type main effect in Experiment 3 showed a disparate pattern from Experiments 1 and 2. One possibility is that color stimuli are inherently robust in saliency so as not mitigated by repetition, rendering the saliency difference between the target and the reference negligible. Another possibility is that the color stimuli differ from one another by more than one psychological dimension (hue and saturation here, unlike shape or orientation only in the previous two experiments). Analogous to the failure for conjunctive features to pop out in visual search, the oddball paradigm here can be regarded as a temporal version of visual search that is substantially harder. That is, either way, the brain may not have an advantage to spot an oddball among colors based on saliency. Future studies can 1) examine whether our conclusion in Experiments 1 and 2 holds for other stimulus dimensions, and 2) further explore the idea of saliency in temporal visual search, just as in the oddball paradigm, to fully discover the properties of spatio-temporal saliency.

## General discussion

### Isolating top-down attention

Since the initial report of the oddball chronostasis phenomenon [2], the attention [2, 9] and the repetition suppression hypotheses [10–13] have been separately proposed but never directly contrasted in the same experiment. Starting from theoretical analysis, we have decomposed these two hypotheses into four putative factors, and then regrouped them into three testable categories—top-down attention, saliency/adaptation, and prediction error. Empirically, our Experiments 1 and 2 together have shown that top-down attention and saliency/adaptation are the two necessary and equal contributing underlying factors of oddball chronostasis. Experiment 3 has shown that top-down attention alone is a sufficient cause. In sum, our findings suggest that top-down attention is an inherent factor underlying oddball chronostasis. The additional contribution from saliency or adaptation depends on the stimulus dimension. In other words, oddball chronostasis occurs at least partly because the oddball is a target, which has nothing to do with its being odd in the context.

### Saliency vs. adaptation

Among the three remaining hypotheses- saliency, adaptation, and prediction error, prediction error has been consistently ruled out both in our study and Cai et al. [10]. In our Experiments 1 and 2, there was no significant difference between the ordered and the random sequence conditions, which applied to higher-level stimuli such as digits, as well as lower-level properties such as orientations. Although in our Experiment 3, there was not an ordered condition, the insignificant difference between the repeated and the random conditions still suggested that prediction error has no role for color stimuli. In experiment 3 of Cai et al. [10], there was no significant difference between the expected and unexpected digit conditions either. Therefore, in this sense, the ‘surprise’ element is not a critical factor.

The evidence so far may favor the saliency over the adaptation account, but does not entirely rule out the latter one. The results of Experiment 3 can be easily explained by saliency rather than adaptation (see Results for details). This is supported by the discovery of Schindel et al. (see experiment 2 of [13]) that given the luminance of standards was fixed, a dimmer oddball elicited stronger chronostasis than a brighter oddball, which was against the prediction of the adaptation hypothesis. On the other hand, the results in experiment 4 of Cai et al. [10] showed that only when a bar is presented at the original location with the same orientation, chronostasis occurs, which is more consistent with the adaptation hypothesis. Either way, our experiment results have isolated the top-down attention factor, and narrowed down the uncertainty between attention and repetition suppression to a bottom-up source, be it saliency, adaptation, or both.

### Origin of the serial position effect

Our data also contribute to resolving a side issue—the serial position effect, where a later oddball appears to last longer in a sequence. In the literature, two separate theories were proposed. The repetition suppression hypothesis [12] explains the effect by the number of same items before the target, while the temporal preparation hypothesis [16] explains the effect by the accumulative anticipation of the target onset in a sequence. However, a decisive hypothesis testing has not been performed earlier. Specifically, the repetition suppression hypothesis would predict the same CM no matter the serial position if the sequence were a non-repeated one (such as ordered or random). On the other hand, the temporal preparation hypothesis predicts larger CM with later serial positions no matter the sequence type. In our study, by manipulating target serial order orthogonally to sequence type, the interaction effect was not significant across all three experiments. Specifically, non-repeated sequences still showed significant target serial position effect in Experiments 1 and 2, but not in Experiment 3. Therefore, part of the evidence in our study directly supports the temporal preparation hypothesis. It is conceivable that temporal preparation could dominate the serial position effect when the specific stimulus items (such as digits and orientations) are natural and easy to remember, while repetition suppression could prevail when the specific stimulus items are more unfamiliar to the participants. Future studies may pursue whether that is really the case. In sum, our study has systematically teased apart three separate sources of oddball chronostasis—top-down attention, saliency/adaptation, and temporal preparation.

### Relation to other types of chronostasis

Our finding that top-down attention may be an inherent component in oddball chronostasis is closely related to all types of chronostasis, such as chronostasis elicited by the first item, the last item [1], gaze shift (e.g., [4]), and action preparation [5]. If researchers were to identify the contributing factors underlying each type of chronostasis, it is beneficial to isolate the top-down attention part first and let it serve as a baseline, such as the role of the random sequence condition in our experiments (c.f. [22]).

### Alternative interpretations

Although the explicit prediction error component has been excluded, there may still be room for expectation or predictive coding (in the likelihood prediction sense). In a few oddball chronostasis studies, when multiple oddball types are interleaved, the higher likelihood oddball type could positively [10] or negatively [16] modulate chronostasis. Similar mixed results were found in other contexts. With a two-stimulus paradigm, Ulrich, Nitschke, and Rammsayer [23] found that a lower likelihood target elicited stronger chronostasis; by contrast, Matthew and colleagues [24–25] found that lower likelihood novel target induced less or no chronostasis. Since likelihood manipulation is not our main theoretical concern, we will leave this for future investigations.

Schindel and colleagues [13] have advocated the predictive coding view to explain oddball chronostasis and proposed that attention is only drawn toward the oddball because of its lower likelihood of being presented. However, our main finding that a random sequence with 100% certainty target still elicited substantial chronostasis refutes this claim. Top-down attention may operate independently of predictive coding in perceived duration. Needless to say, our discovery resonates with how attention orienting prolongs time perception in general. For instance, exogenous (e.g., [26–27]) and endogenous (e.g., [28]) spatial attention orienting, as well as exogenous and endogenous temporal attention orienting (e.g., [29–30]) both lengthen time perception.

An alternative to the attention hypothesis is that the target elicits greater arousal than the other items. Initially, Pariyadath and Eagleman [11] have demonstrated in their experiment 3 that using an emotionally salient stimulus did not make oddball chronostasis stronger. Although one may argue that it could be due to ceiling effect, in light of the findings of Cai et al. [10] and our Experiments 1 and 2, the ‘surprise’ element (ordered vs. random, see green bars vs. blue bars in Figs 3F and 4F), which presumably would also have elevated arousal, did not in turn modulate CM. Therefore, the arousal hypothesis is overall less likely.

### Neurophysiological and computational implications

Resolving the debate over attention vs. repetition suppression and over temporal preparation vs. repetition suppression (see all likely scenarios in Fig 2) is critical to identifying the roles of underlying neural mechanisms. Qualitatively, our study has experimentally dissociated three separate factors that give rise to oddball chronostasis—top-down attention, saliency/adaptation, and temporal preparation. Quantitatively, the relative contribution ratio of top-down attention to saliency/adaptation is essential to computational modeling of perceived duration. We have demonstrated that it could be close to 1:1 or 1:0 depending on the stimulus dimension (digits, orientations, or color). The experimental design can thus be utilized in future neural imaging studies to pin down the neural correlates of each source. Since many flavors of neural models have been proposed (for an overview, see [31]), it remains to be seen whether these three top-down and bottom-up sources of human duration perception modulators converge onto the same network node to determine perceived duration. If this were the case, the sensory cortex neural response amplitude hypothesis advocated by Eagleman and colleagues [10, 12, 17, 32] might be one interesting possibility. If not, a core timing network which consists of the supplementary motor area and the basal ganglia along with other context-dependent nodes [33] could be separately regulated by these three disparate factors. The answers await future investigations.

## Conclusions

1. This is the first study that directly contrasts the attention with the repetition suppression theories, and the temporal preparation with the repetition suppression accounts. The discoveries are crucial to understanding the mechanisms underlying how time is perceived in a context.
2. Our experiments tested three categories of theoretical components to resolve the controversy between the attention [2, 9] and the repetition suppression theories [10–13] of oddball chronostasis: top-down attention, saliency/adaptation, and prediction error.
3. Results suggest that top-down attention is sometimes sufficient (to isoluminant colors) and the other sometimes necessary (to digits and orientations) to explain oddball chronostasis. In the latter case, it explains about half of the effect; saliency/adaptation explains the other half. Prediction error does not play a critical role.
4. Between the temporal preparation [16] and the repetition suppression hypothesis [12, 17], the data with digits and orientations favor the former because non-repeated (ordered or random) sequences still exhibit target serial position effects. In this case, prior repetitions are not crucial to render a later target appear lasting longer.
5. Therefore, oddball chronostasis has two important underlying contributors unrelated to event oddness or unexpectedness—top-down attention and temporal preparation; sometimes bottom-up saliency/adaptation co-contributes.
